# Diagnosis and treatment experience of cecal diverticulitis in six pediatric patients

**DOI:** 10.3389/fped.2024.1478296

**Published:** 2024-12-12

**Authors:** Xiao Li, Wei Liu, Shisong Zhang, Hongzhen Liu

**Affiliations:** ^1^ Department of General Surgery, Children’s Hospital Affiliated to Shandong University, Jinan, China; ^2^ Department of General Surgery, Jinan Children’s Hospital, Jinan, China

**Keywords:** cecal diverticulitis, children, clinical characteristics, diagnosis, treatment

## Abstract

**Objective:**

This study aims to summarize the clinical characteristics, diagnostic methods, and treatment experience of cecal diverticulitis in children.

**Method:**

The clinical data of six pediatric patients with cecal diverticulitis, treated at Children's Hospital Affiliated to Shandong University from November 2021 to May 2023, were retrospectively analyzed.

**Result:**

All patients presented with abdominal pain primarily in the lower right abdomen. Two cases had fever with a maximum body temperature not exceeding 38.0°C. Three cases exhibited elevated inflammatory markers such as white blood cell count and C-reactive protein (CRP) upon admission. Three children were misdiagnosed with acute appendicitis based on preoperative color Doppler ultrasound. Two children were treated with third-generation cephalosporins and ornidazole for anti-inflammatory therapy and were cured after 6 and 9 days of hospitalization, respectively. Four children underwent laparoscopic surgery with excision of the cecal diverticulum and cecal repair, all of whom recovered well without postoperative complications. The operation duration ranged from 100 to 170 min, with an average of 140 min. Intraoperative blood loss ranged from 5 to 10 ml, averaging 6.75 ml. The overall length of hospital stay was 8–12 days, with an average stay of 9.5 days. All patients were followed up until December 2023, with no recurrences observed.

**Conclusion:**

Children with cecal diverticulitis, especially complex cecal diverticulitis, are easily misdiagnosed as acute appendicitis. Acute simple cecal diverticulitis can be treated with anti-inflammatory therapy. Laparoscopic cecal diverticulectomy combined with cecal repair is a feasible and effective method for treating acute complex cecal diverticulitis.

## 1 Introduction

Cecal diverticulitis refers to the presence of a diverticulum-like structure in the cecum, accompanied by visible inflammation (1). When the lesion is limited to cellulitis around the diverticulum, it is classified as acute simple diverticulitis. As the disease progresses, leading to local abscess formation, diverticulum perforation, bleeding, intestinal obstruction, fistula formation, diffuse peritonitis, and other complications, it is classified as complex cecal diverticulitis (2, 3). Most cecal diverticula are acquired (4), and their pathogenesis (5, 6) is thought to involve the penetration of straight arterioles into the colonic annulus muscle, forming a relatively weak area. Increased pressure in the intestinal cavity causes the mucosal and submucosal layers to protrude outward, forming diverticula. Prolonged retention of feces in the diverticulum can lead to bacterial overgrowth and toxin production. Congenital cecal diverticulitis is an inflammation of the diverticula, which formed by the protrusion of the entire intestinal wall, including the muscular layer. Cecal diverticulitis is easier to diagnose in adults, but it is rarely reported in children and is often misdiagnosed as acute appendicitis. Therefore, it is necessary to enhance pediatricians’ understanding of the disease and master its diagnosis and treatment (7). This paper aims to summarize the experiences and lessons in diagnosing and treating cecal diverticulitis in children and to explore the optimal treatment methods.

## 2 Clinical data and methods

### 2.1 General data

**Figure 1:**
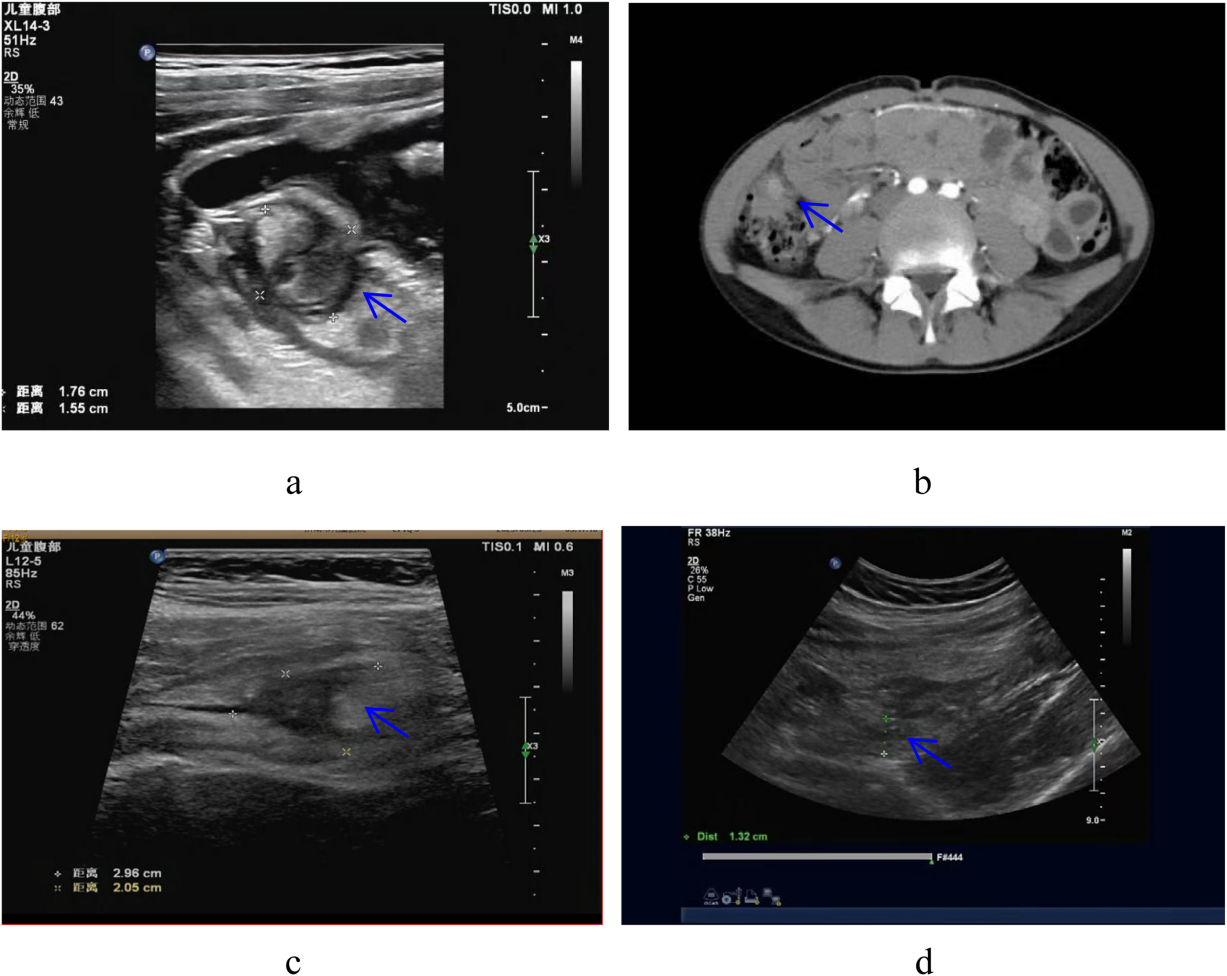
**(a,b)** in children with simple cecal diverticulitis, inflammatory mass can be seen under ultrasound and CT (→ mark). **(c,d)** In children with complex cecal diverticulitis was misdiagnosed as acute appendicitis under ultrasound. Ultrasound and CT scans follow standardized protocols and are observed jointly by two senior specialists. When there is disagreement, the differences between observers are discussed through consultation to minimize differences between observers.

There were six children in this group, including four males and two females. The age ranged from 4 years to 12 years and 5 months, with an average of 8 years and 5 months. All cases presented with abdominal pain primarily in the right lower abdomen; two cases had fever, with the highest body temperature less than 38.0℃. Nausea and vomiting were observed in two cases, and the disease course ranged from 1 to 15 days. White blood cell counts ranged from 5.2 to 22.26 (×10^9^/L); neutrophil ratios from 31.4% to 88.3%; and C-reactive protein levels from 5.62 to 192.41 mg/L. Before admission, all six cases underwent ultrasonography. Three cases showed inflammatory masses in the lower right abdomen and were diagnosed with cecal diverticulitis, while the other three cases were misdiagnosed with acute appendicitis (see Table 1, Figure 1). This study was reviewed and approved by the Ethics Committee of the Children's Hospital Affiliated to Shandong University, and obtained parents’ informed consent.

**Table 1:** General information of cecal diverticulitis in six pediatric patients.

Case	Age (year)	Course of disease (day)	Temperature (°C)	Weight (kg)	Constipation (Y/N)	WBC (10^9^/L)	Neut%	CRP (mg/L)	Ultrasonography
1	8.2	2	Normal	22	N	5.2	31.4	10.8	Cecal diverticulitis
2	11.3	2	Normal	31	N	11.7	69.9	5.62	Cecal diverticulitis
3	4.1	15	Normal	18.5	Y	8.6	61.4	7.6	Cecal diverticulitis
4	12.2	1	Normal	79	Y	22.26	87.7	192.4	Acute appendicitis
5	12.4	2	≤37.6	59	Y	13.8	88.3	32.1	Acute appendicitis
6	10.6	3	≤37.8	56	N	15.7	80.5	25.6	Acute appendicitis

### 2.2 Treatment methods

#### 2.2.1 Treatment of simple cecal diverticulitis

There were three cases of acute simple cecal diverticulitis, two (cases 1 and 2) of which were treated conservatively with third-generation cephalosporins and ornidazole/metronidazole. One case (case 3) required surgical intervention after a prolonged disease course of 15 days and unsuccessful conservative treatment. During the operation, a diverticule-like protrusion was found on the lateral side of the cecum, about 1.1 × 1.0 × 0.8 cm in size. A wedge resection was performed 2 mm outside the diverticular mass. The intestinal wall was repaired with 4–0 absorbable sutures. The appendix was also removed.

#### 2.2.2 Treatment of complex cecal diverticulitis

**Figure 2:**
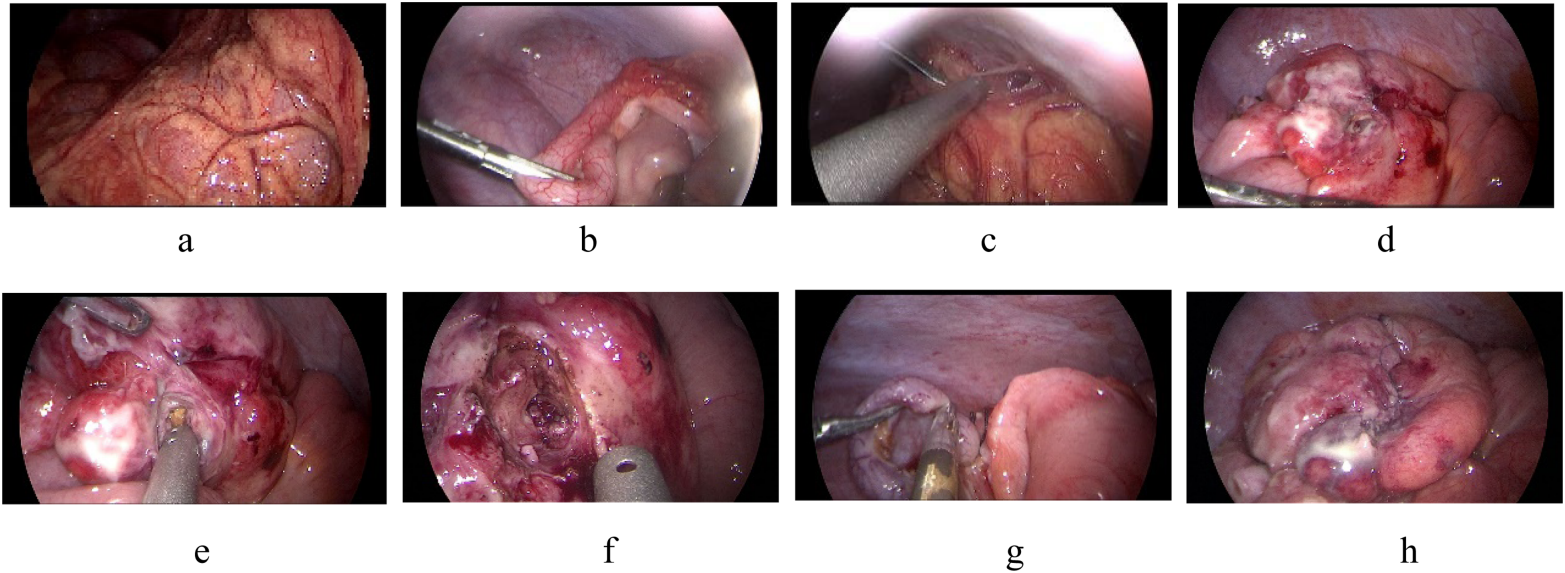
**(a)** The right lower abdominal intestinal is surrounded by omentum, **(b)** the intestinal and omentum were adherent to the abdominal wall, **(c)** the appendix showed obvious redness and swelling, **(d)** the cecum has a diverticulum with perforation, **(e)** fecalith in the diverticulum, **(f)** remove necrotic tissue around the diverticulum, **(g)** resect the appendix, **(h)** repairing the colon.

Three cases of complex cecal diverticulitis (cases 4, 5, and 6) underwent laparoscopic surgery. During the operation, the appendix was examined, but no obvious suppuration, gangrene or perforation was observed. The appendix found during operation was inconsistent with the clinical symptoms, examination and laboratory results of the child. Further exploration showed that the diverticulum (about 1.0–1.5 cm in size) was visible in the medial part of the cecum, and the root perforation of the diverticulum was visible in all 3 cases. The cecal diverticula was removed and the colon was repaired with a 4–0 absorbable suture. Finally, the appendix was removed and a drainage tube was placed in the pelvic cavity (see Figure 2).

### 2.3 Observational indicators

For conservatively treated patients, the primary observational Indicators included hospitalization duration, treatment efficacy, and recurrence rate. For surgically treated patients, additional observations included operation time, intraoperative blood loss, Postoperative eating time, incidence of wound infection, and occurrence of intestinal fistulas.

### 2.4 Patient follow-up

Patients were followed up by outpatient or telephone follow-up. Using the patient's discharge date as a starting point, the patient was asked whether he had recurrent symptoms and his treatment status after discharge. The follow-up method was ultrasound, and abdominal CT and colonoscopy were performed when necessary. All patients were followed up until December 2023.

## 3 Results

**Figure 3:**
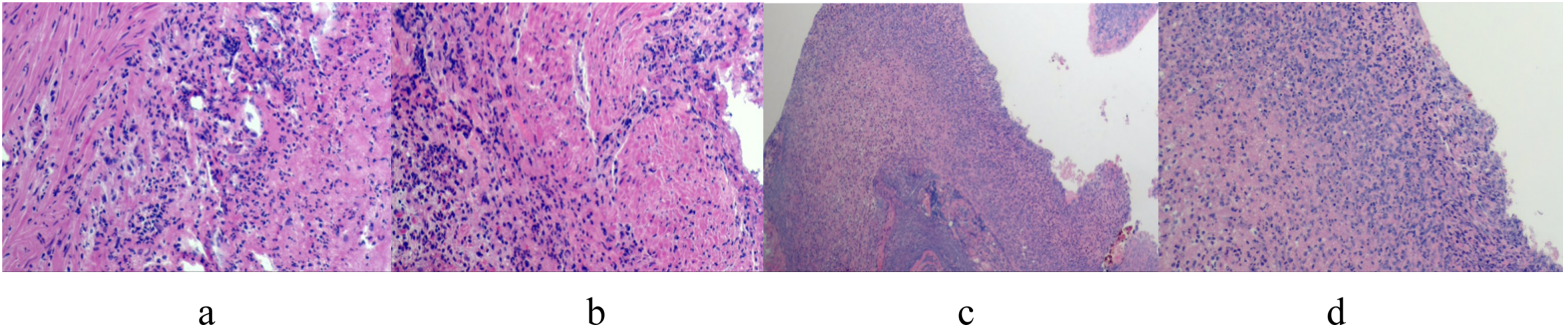
**(a–d)** postoperative pathologic findings: large number of neutrophil infiltration in the diverticulum wall, consistent with diverticulitis changes.

Two patients treated conservatively with third-generation cephalosporins and ornidazole recovered and were discharged after 6 and 9 days, respectively, with no recurrence upon follow-up at 12 and 3 months. The other four patients underwent laparoscopic surgery, with the operation time ranging from 100 to 170 min and an average of 140 min. Intraoperative blood loss ranged from 5 to 10 ml, averaging 6.75 ml. The postoperative recovery time of gastrointestinal function was 3–7 days, with an average of 5.5 days. The extraction time of the pelvic drainage tube was 3–8 days, with an average of 6.5 days. The hospital stay ranged from 8 to 12 days, averaging 9.5 days. (Case No.3–6 are children undergoing operation. The intraoperative and postoperative conditions are shown in Table 2). No postoperative complications such as incision infection, residual abdominal infection, or intestinal leakage were observed. All patients were cured and discharged from the hospital. Postoperative pathological findings included cecal diverticulitis, simple appendicitis, or suppurative appendicitis (see Figure 3). Follow-up the 4 patients for 2–22 months post-operation, averaging 14 months, revealed no recurrence and good general condition.

**Table 2:** Intraoperative and postoperative conditions of children undergoing surgery.

Case	Type	Operation time (min)	Postoperative pathology	Recovery time of gastrointestinal function (day)	Extraction time of the drainage tube (day)	Hospital stay (day)
3	Simple	100	Cecal diverticula, acute and chronic inflammation. Neutrophils were found in the vascular lumen of the appendix wall.	3	5	8
4	Complex	160	Cecal diverticulitis with perforation. Acute suppurative appendicitis.	6	6	8
5	Complex	170	Cecal diverticulitis with perforation. Acute suppurative appendicitis.	7	8	12
6	Complex	130	Cecal diverticulitis with perforation. Acute simple appendicitis.	6	7	10

## 4 Discussion

The incidence of colonic diverticulosis in China ranges from 0.2% to 1.9%, and less than 5% of cases leading to secondary diverticulitis, mainly in the right colon (8). With the widespread application of imaging examinations and the popularization of colonoscopy and laparoscopy, the diagnosis rate has been gradually increasing. Acute cecal diverticulitis in children is rare and often misdiagnosed as acute appendicitis or appendagitis even a tumor, due to similar clinical presentations and abdominal symptoms. When the course of the disease is long and the intraoperative local adhesions are severe, these diseases are also difficult to identify intraoperatively. In this study, three cases were misdiagnosed as appendicitis, and these three misdiagnosed cases were complex cecal diverticulitis, so we can see that complex cases are more likely to be misdiagnosed.

It is difficult to diagnose children's cases solely according to clinical symptoms, abdominal signs and B-ultrasonography. The accuracy of abdominal ultrasound for diagnosing this disease in adults exceeds 90% (9). However, in this study, only three cases were correctly diagnosed preoperatively, and all were simple cecal diverticulitis, which is related to the low incidence of this disease in children, children's uncooperation in physical examination and auxiliary examination, and doctors' insufficient knowledge of this disease. Abdominal CT has high sensitivity and specificity for diagnosing cecal diverticulitis and identifying complications (10). If the diagnosis is difficult, CT should be performed. When a child is diagnosed with acute appendicitis, conservative anti-inflammatory therapy is effective for simple cecal diverticulitis, and for the other part of children, most of them underwent laparoscopic surgery. If intraoperative exploration found that the degree of appendix lesions is inconsistent with the preoperative examination results and the child's symptoms and signs, we usually perform a routine exploration of the small intestine, If no positive findings, the colon should be carefully explored, which is helpful to detect the Cecal diverticulitis in time.

The treatment of acute cecal diverticulitis is controversial. Some scholars argue that acute simple cecal diverticulitis is non-infectious and does not require anti-inflammatory treatment (11). However, the latest guidelines recommend conservative treatment with antibiotics for symptomatic simple cecal diverticulitis. For patients with poor conservative response, repeated recurrence of colonic diverticulitis or immunosuppression, surgical treatment can be chosen (12). Combining the cases in this study, all the children patients had abdominal pain symptoms, and anti-inflammatory treatment could significantly alleviate the symptoms. This option is more reasonable. In this study, there were three children with simple cecal diverticulitis, two patients were discharged after conservative anti-inflammatory treatment, and one patient was treated by surgery due to a long history of the disease and poor conservative treatment effect. Compared with complicated cecal diverticulitis cases, the local adhesion was lighter, the operation time was shorter, the intraoperative bleeding was less, and the recovery was faster.

For children with complex caecal diverticulitis, surgeons prefer surgical treatment (13, 14). The choice of surgical methods is determined by the location, number and complications of diverticulum observed during surgery. For the patients with mild inflammatory lesions around the diverticulum, surgeons often resect the diverticula completely and suture the intestinal wall and seromuscular layer intermittently. The advantage of this surgical method is to retain the structure and function of the ileocecal valve and have little effect on the growth and development of children. The disadvantage is an increased risk of intestinal leakage and secondary operation. If intraoperative exploration finds large cecum perforation, severe intestinal wall inflammation, or serious abdominal cavity contamination, patients are usually not suitable for first-stage laparoscopy surgery, because the operation is really difficult, the operation time is prolonged, and the risk of anesthesia is increased (15, 16). We have three children with complex cecal diverticulitis who performed laparoscopic surgery, which is closely related to the experience and skills of the surgeon. In addition to cecal diverticulectomy, appendectomy was also performed in all the children, because the inflammation of the appendix at this time was usually caused by cecal diverticulitis. In order to avoid repeated operations for appendicitis in the future, Simultaneous appendectomy was more beneficial for the children. However, in laparoscopic appendectomy, if simple cecal diverticulitis is found, it is generally not removed, because anti-inflammatory treatment is effective, and the removal of diverticulum is more traumatic for children.

The author observed the cases in this group, children with simple cecal diverticulitis, mainly presented abdominal pain, not serious, inflammatory markers and temperature are basically normal; the heat peak of fever with complex cecal diverticulitis is lower than that in appendicitis. Cecal diverticulitis is rare in children, and there were only 6 cases in this study, with a small sample size, so our clinical research still needs to be further in-depth.

To sum up, acute cecal diverticulitis in children is rare and often difficult to distinguish from acute appendicitis. Conservative antibiotic treatment is effective for acute simple cecal diverticulitis. Complex cecal diverticulitis should be treated with surgery as soon as possible. Laparoscopic surgery is safe and effective for treating pediatric acute cecal diverticulitis and merits further promotion. It is hoped that the study will increase clinicians' awareness of differential diagnosis and treatment when they encounter similar cases in the future.

## Data Availability

The datasets presented in this study can be found in online repositories. The names of the repository/repositories and accession number(s) can be found in the article/Supplementary Material.
